# An Unusual Case of Angiotensin-Converting-Enzyme Inhibitor-Related Penile Angioedema with Evolution to the Oropharynx

**DOI:** 10.5811/westjem.2015.8.28061

**Published:** 2015-11-18

**Authors:** Jonathan G. Wagner, Elias M. Bench, Lee Plantmason

**Affiliations:** Keck School of Medicine of the University of Southern California, Los Angeles County + University of Southern California (LAC+USC), Department of Emergency Medicine, Los Angeles, California

## Abstract

A 52-year-old African American male with a long history of poorly controlled hypertension presented to the emergency department (ED) with two days of genital edema and pain. During ED work-up, the patient developed sudden onset of non-pitting, non-pruritic, and non-urticarial upper lip edema. Review of his antihypertensive medication list revealed that he normally took benazepril, highly suggestive of a diagnosis of angiotensin-converting-enzyme inhibitor-related angioedema (ACEI-RA). We present the first reported case of penile ACEI-RA that progressed to involve the oropharynx. The ED management of the condition and some of the newer treatment options available for ACEI-RA is also briefly discussed.

## INTRODUCTION

Angioedema, one of the true airway emergencies, is a non-pitting, often asymmetric swelling of subcutaneous or submucosal tissues. Typically isolated to an oropharyngeal distribution, it can also affect the gastrointestinal tract, extremities, and genitalia.^1^ Angiotensin-converting-enzyme inhibitor-related angioedema (ACEI-RA) is the most common type of angioedema and can occur in both long- and short-term use of angiotensin-converting-enzyme inhibitor (ACEI) medications.^1^ Though ACEI-RA is well-described in the literature and not a rare disorder, angioedema of the penis has only been reported in three previous publications (four total cases overall).^2^–^4^ In each case, the symptoms were isolated to the genitalia without additional sites of involvement. Here we present the first known case of a patient with delayed-onset ACEI-RA that was initially isolated to the genitalia with evolvement to the oropharynx, and discuss possible etiologies and treatment options for emergency department (ED) management.

## CASE REPORT

A 52-year-old African-American male with a past medical history of poorly controlled hypertension (due to intermittent medication compliance), alcohol abuse, and alcohol withdrawal was referred to the ED for two days of penile and scrotal edema and pain. The patient stated that he noted the non-pruritic, non-erythematous diffuse genital swelling two days prior, but had delayed seeking treatment as he assumed it would self-resolve. Due to increasing pain, he visited his primary care practitioner (PCP), who then immediately referred him to the ED for further evaluation. On interview, he denied recent sexual intercourse, allergies to latex/condoms, penile rings, painful erections, lotions or creams applied to the genital area, traumatic sexual injuries, penile discharge, or history of sexually transmitted infections. Additionally, the patient also denied any other new exposures, known allergies, history of similar symptoms that would suggest angioedema to the face, extremities, or genitals, or a family history of recurrent angioedema. The patient stated that he only knew the name of two of his five prescribed antihypertensive agents, hydrochlorothiazide and benazepril, both of which he had taken for at least 10 years. His benazepril dose had recently been increased from 20mg to 40mg, but he admitted to not taking any of his anti-hypertensive medications on the day of his ED visit. He also disclosed a daily average alcohol intake of “one gallon of vodka per day” and had consumed one pint that morning. A review of systems was negative for any other complaints, including fevers, chills, dysuria, hematuria, urinary retention, abdominal or back pain, respiratory symptoms, any sensation of facial, oral, or pharyngeal swelling, change in voice, or difficulty swallowing.

On physical examination, blood pressure was significantly elevated at 198/112mmHg, pulse was 87 beats per min, respiratory rate was 20 breaths per min, oral temperature was recorded at 98.4°F, and room air pulse oximetry saturation of 97%. His skin exam revealed no rashes, urticaria, or discolorations. The facial, oropharyngeal, cardiopulmonary, and abdominal examinations were within normal limits. Examination of the genitalia revealed edema isolated to the scrotum and uncircumcised penis. The edema was soft and non-pitting with no excessive warmth, erythema, induration, or other signs of local infection. The foreskin was partially retractable with no evidence of paraphimosis or balanoposthitis. The bladder was non-distended, there were no hernias, and scrotal contents were of normal size and consistency with no epididymal tenderness. There was no penile discharge or inguinal lymphadenopathy. The extremities did not exhibit any edema, erythema, or skin changes.

ED work-up revealed a normal urinalysis with no hematuria, proteinuria, or evidence of infection. The patient’s complete blood count, comprehensive metabolic panel, and prothrombin time were within normal limits and did not exhibit evidence of hypoalbuminemia, increased creatinine, or impaired hepatic function. The patient also did not exhibit any symptoms or physical exam findings suggestive of congestive heart failure, although an incidental right middle lobe pulmonary nodule was noted on the patient’s chest radiograph. The preliminary read of the testicular ultrasound described a small hydrocele, but no evidence of torsion, abscess, tumor, or varicocele. The patient’s blood pressure improved to 170/98 with subsequent stay in the ED and upon restarting his home dose of hydrochlorothiazide.

While awaiting the formal read of his scrotal ultrasound, the patient developed anterior oropharyngeal edema isolated to his upper lip. On examination, there appeared to be no airway involvement and the patient denied change in voice, difficulty swallowing, or shortness of breath. Given the acute onset of oropharyngeal edema, the patient was given diphenhydramine 50mg IV, famotidine 40mg IV, and methylprednisolone 125mg IV for possible allergic reaction versus acute onset of angioedema. He was observed in the ED for six hours, without progression or significant improvement in symptoms, and discharged home with explicit instructions to discontinue his benazepril and to avoid all ACEIs and angiotensin receptor blockers (ARBs). It was recommended that the patient follow up with his PCP as soon as possible for medication reconciliation to optimize his blood pressure control and to return to the ED for any of the following symptoms: increased oropharyngeal swelling, respiratory difficulty, difficulty urinating, worsening penile pain, or paraphimosis. Four days later, he followed up with his PCP and his symptoms had completely resolved.

## DISCUSSION

ACEI-RA was first reported in 1984^5^ and is now the leading cause of drug-induced angioedema, accounting for up to 30% of angioedema cases presenting to the ED.^6^ The incidence of angioedema in patients taking ACEI has been estimated at 0.68%,^7^ most commonly affecting the face, lips, tongue, and upper airway. Infrequently, it involves the gastrointestinal tract, extremities, and very rarely, the genitalia.^1^ While the majority of cases of ACEI-RA present within the first week of exposure, many patients will experience symptoms after years of ACEI therapy.^8^, ^9^ The most important risk factor for ACEI-RA is African descent, followed by previous angioedema episode, age >65 years, nonsteroidal anti-inflammatory drug (NSAID) use, female sex, history of drug-related rash, and seasonal allergies.^1^ Angioedema is divided into mast-cell mediated and bradykinin-associated angioedema (ACEI-RA), with a lack of pruritus or urticaria as the hallmark of bradykinin-associated angioedema.

A clinical diagnosis of ACEI-RA should be considered in a patient with angioedema to a characteristic anatomic site, without pruritus or urticaria, and a history of ACEI exposure. In all patients who present to the ED with swelling to an affected area, it is paramount to differentiate angioedema from other causes of soft-tissue swelling (i.e. cellulitis, contact dermatitis, low oncotic states, etc.). ACEI-RA should be distinguished from mast-cell associated angioedema as ACEI-RA is minimally responsive to antihistamines, glucocorticoids, epinephrine, and lacks allergic symptoms.^10^

The mainstay of treatment for ACEI-RA, no matter the affected location, is the prompt discontinuation and future avoidance of all ACEIs, assessment of the airway, and supportive care. In those with suspected oropharyngeal involvement, the airway should be evaluated emergently, as up to 10% of patients will require intubation.^11^ In patients with progressive symptoms or those who will require advanced airway management imminently, medical management should be attempted. Icatibant, a synthetic bradykinin B2 receptor antagonist, has been shown to be effective for severe ACEI-RA if given within the first 10 hours of symptom onset.^12^ Medications approved for hereditary angioedema should also be considered in severe cases of ACEI-RA in which intubation is imminent. These include fresh frozen plasma, purified C1 inhibitor concentrate, and ecallantide.^1^ Patients with ACEI-RA should be given strict precautions to avoid all ACEIs in the future, as continued use is associated with increased recurrence and severity.^13^ The use of ARBs is controversial, as patients with ACEI-RA have a 1.5–10% risk of recurrent angioedema when switched to ARBs.^14^, ^15^ Those with penile angioedema should be instructed to avoid retraction of the foreskin to reduce the risk of paraphimosis.^3^

Importantly, as seen in our case, penile angioedema can be associated with oropharynx involvement, and ED physicians should perform a thorough examination of the upper airway and educate their patients on the possibility of this potentially life-threatening complication. Patients in whom there is no disease progression, no involvement of the larynx, tongue, or elevation of the floor of the mouth can be discharged safely if they express understanding of the possible complications of ACEI-RA, have the ability to return promptly if necessary, and have follow up with a primary provider.

This case report, as well as the three previous publications describing genital angioedema as a presentation of ACEI-RA, demonstrates the importance of including angioedema in the differential for new onset penile or scrotal swelling. This case is the first to show that isolated genital angioedema can progress to involve the oropharynx, further underscoring the importance of airway evaluation in all patients in whom ACEI-RA is suspected and observation to ensure there are no additional complications. Following diagnosis, physicians can reassure patients that they are experiencing a drug reaction that is likely to resolve quickly without long-term complications, reducing significant anxiety and distress associated with acute swelling of the genitalia.
